# Crystals on the Iris: Russell Bodies in Fuchs Uveitis

**DOI:** 10.18502/jovr.v16i3.9446

**Published:** 2021-07-29

**Authors:** Emanuela Interlandi, Francesco Pellegrini, Erika Mandarà, Carlos Pavesio

**Affiliations:** ^1^Department of Ophthalmology, Ospedale del Mare, Naples, Italy; ^2^Department of Ophthalmology, “Santo Spirito” Hospital, Pescara, Italy; ^3^Department of Ophthalmology, “Maria Paternò Arezzo” Hospital, Ragusa, Italy; ^4^Moorfields Eye Hospital, NHS Foundation Trust, London, UK

##  PRESENTATION

**Figure 1:**
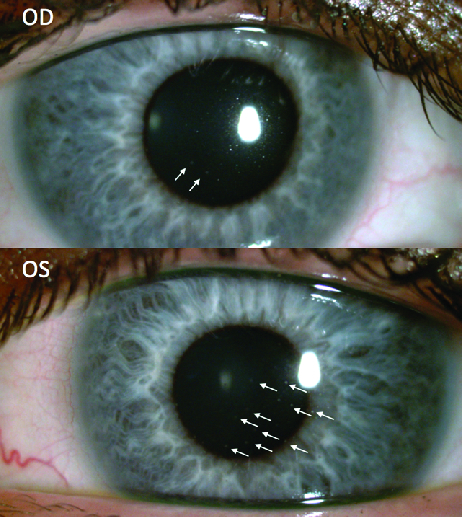
Endothelial stellate keratic precipitates (OS > OD) (white arrows).

**Figure 2:**
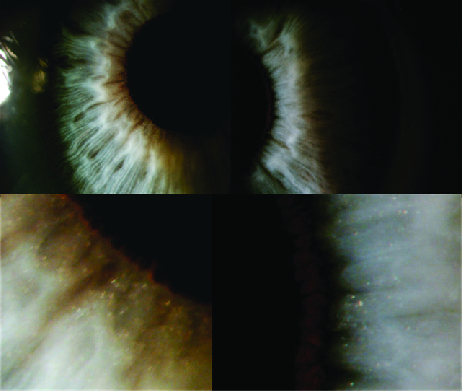
Minute crystals on the iris surface at great magnification of iris OU.

**Figure 3:**
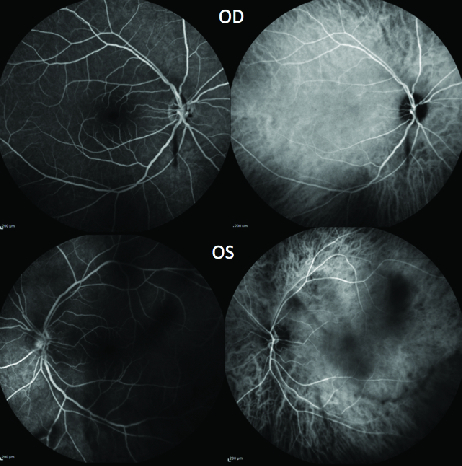
FA and ICG showing dark hypofluorescent mobile spots of vitritis (OS > OD).

**Figure 4:**
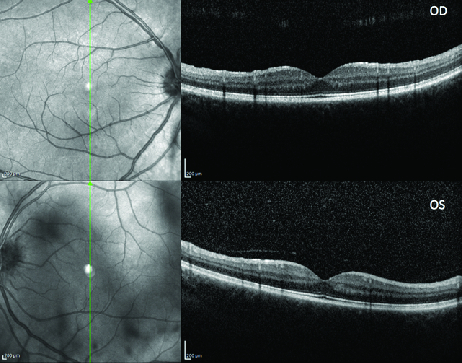
SD-OCT showed vitreous hyper-reflectivity OS.

A 32-year-old otherwise healthy female with a history of bilateral chronic vitritis was sent to our center for a second opinion. She denied major illness, drugs intake, or previous trauma. Visual acuity was 20/30 in both eyes (OU) and intraocular pressure was 15 mmHg. Slit lamp examination revealed small stellate keratic precipitates [Figure 1] throughout the entire extent of corneal endothelium and minute crystals on the iris surface in both eyes [Figure 2]. Funduscopic examination showed a mild vitritis with no signs of retinal vasculitis OU. The patient underwent fluorescein angiography (FA) and indocyanine green angiography (ICG), both with unremarkable results except for dark hypofluorescent mobile spots consistent with vitritis [Figure 3]. Optical coherence tomography did not show macular involvement [Figure 4]. FA showed. Laboratory tests were performed to exclude infectious or immune-mediated causes of uveitis together with serum protein electrophoresis and resulted in range. She was diagnosed with bilateral Fuchs' uveitis (FU). FU is a low-grade, chronic uveitis of unknown origin. Less than 10% of cases are bilateral.^[1]^ The highly refractile deposits (termed Russell bodies) are rare biomicrosopic findings in eyes with FU.^[2]^

In 1969, the first case of iris crystals was described in a patient with chronic uveitis.^[3]^ The clinical appearance of iris crystals is that of highly refractile minute bodies that glisten with illumination at the slit beam set at different angles. An eye may have from one to numerous iris crystals, and their number may change from time to time.^[4]^ Russell bodies occur in many types of chronic inflammation including chronic uveitis and various B lymphocyte tumor lines, however, they are more common in FU. Among the 24 patients with Russell bodies, 17 (71%) had FU, 6 (25%) had idiopathic anterior or posterior chronic uveitis, and one (4%) had Behçet's disease. However, none of them had bilateral iris crystals, and the authors concluded that “Bilateral crystals appear to be extremely rare”. These crystals probably represent plasma cells filled with immunoglobulin,^[4]^ and may occur as a result of a block in the normal pathways of Ig secretion within plasma cells. The diagnosis of FU is based on clinical findings as no confirmatory laboratory tests are available. Our patient was diagnosed with FU based on the presence of bilateral chronic uveitis, vitritis, stellate keratic precipitates, and absence of synechiae; she was given no therapy and scheduled for further controls. At two years follow-up, Russell bodies were still present. In conclusion, iris crystals appear to be rare but may be underdiagnosed as they are small and can easily be missed. They are associated with diseases with active immunoglobulin production in the anterior chamber. They are usually unilateral even in cases of bilateral uveitis. The pathophysiology of iris crystals remains unresolved, but their formation is a reflection of plasma cell activity within the iris stroma. Laboratory tests such as serum electrophoresis helps in determining the presence of underlying hypergammaglobulinemia. We recommend a careful search for iris crystals in every case of uveitis to document this remarkable clinical sign.

##  Declaration of Patient Consent

The authors certify that they have obtained all appropriate patient consent forms. In the form the patient has given her consent for her images and other clinical information to be reported in the journal. The patient understand that her name and

initial will not be published and due efforts will be made to conceal his identity, but anonymity cannot be guaranteed.

##  Financial Support and Sponsorship

Nil.

##  Conflicts of Interest

There are no conflicts of interest.
